# Elevated Serum LPS in Newly Diagnosed Hashimoto’s Thyroiditis: A Case–Control Study in Bulgaria

**DOI:** 10.3390/clinpract16020026

**Published:** 2026-01-26

**Authors:** Desislav Tomov, Boryana Levterova, Valentina Mihailova, Dimitar Troev, Zlatina Tomova, Yordanka Uzunova, Maria Orbetzova

**Affiliations:** 1 Research Institute, Medical University of Plovdiv, 120 “Bratya Bukston” Str., 4004 Plovdiv, Bulgaria; desislav.tomov@mu-plovdiv.bg (D.T.); mihailova83@abv.bg (V.M.); zlatina.tomova@mu-plovdiv.bg (Z.T.); yordanka.uzunova@mu-plovdiv.bg (Y.U.); 2 Department of Health Management and Healthcare Economics, Faculty of Public Health, Medical University of Plovdiv, 15-A “Vasil Aprilov” Blvd., 4002 Plovdiv, Bulgaria; 3 Department of Medical Biology, Faculty of Medicine, Medical University of Plovdiv, 15-A “Vasil Aprilov” Blvd., 4002 Plovdiv, Bulgaria; 4 Department of Endocrinology, Faculty of Medicine, Medical University of Plovdiv, UMHAT” St. George” Plovdiv, Clinic of Endocrinology and Metabolic Diseases, 15-A “Vasil Aprilov” Blvd., 4002 Plovdiv, Bulgaria; dimitar.troev@mu-plovdiv.bg; 5 Department of Prosthetic Dental Medicine, Faculty of Dental Medicine, Medical University of Plovdiv, 3, Hristo Botev Blvd., 4002 Plovdiv, Bulgaria; 6 Department of Bioorganic Chemistry, Faculty of Pharmacy, Research Institute, Medical University of Plovdiv, 15-A “Vasil Aprilov” Blvd., 4002 Plovdiv, Bulgaria; 7 Department of Endocrinology, Faculty of Medicine, Medical University of Plovdiv, 15-A “Vasil Aprilov” Blvd., 4002 Plovdiv, Bulgaria; maria.orbetzova@mu-plovdiv.bg

**Keywords:** Hashimoto’s thyroiditis, microbiota, diet, LPS, Bulgaria

## Abstract

**Background:** Hashimoto’s thyroiditis (HT) is a prevalent autoimmune disorder, often diagnosed late due to its asymptomatic or nonspecific presentation. Emerging evidence suggests that gut-derived lipopolysaccharides (LPS) may contribute to autoimmune activation. **Objective**: The primary objective of this study was to assess circulating LPS concentrations and dietary patterns in patients with Hashimoto’s thyroiditis compared to healthy controls. **Methods**: A hospital-based case–control study was conducted involving 105 HT patients and 25 healthy controls. Serum LPS concentrations, thyroid hormone profiles, and autoantibody levels were assessed. Dietary patterns were evaluated using the validated KomPAN questionnaire. **Results**: HT patients exhibited significantly higher serum LPS levels, particularly those with elevated anti-TPO and TRAB antibodies. A positive correlation was found between LPS and the fT3/fT4 ratio (*r* = 0.247, *p* = 0.006), and a negative correlation with fT4 (*r* = −0.314, *p* < 0.001). Dietary analysis revealed lower Pro-Healthy Diet Index scores in HT patients (3.94 vs. 5.34, *p* = 0.001), with increased consumption of processed foods and reduced intake of whole grains and oats. **Conclusions**: Elevated levels of lipopolysaccharides (LPS) and unhealthy dietary patterns may play a role in the development of thyroid autoimmunity. Taken together, these observations are consistent with a multifactorial model that potentially involves gut barrier dysfunction, endotoxemia, and nutritional factors in HT pathogenesis.

## 1. Introduction

The thyroid gland plays a pivotal role in regulating the body’s overall metabolism through the hormones it secretes [1]. Autoimmune thyroid diseases, principally manifesting as Hashimoto’s thyroiditis (HT) and Graves’ disease (GD), are highly prevalent on a global scale and are no longer confined to developed countries [2]. Despite the fact that the aetiology of the condition remains incompletely understood, there is a growing body of research investigating the interactions between genetic predisposition, psychological stress and the gut microbiota. Increasing attention is also being directed towards dietary factors, which are more amenable to modification and may meaningfully contribute to the development of these disorders [3].

In recent years, there has been an increased academic interest in the role of gut microbiota and its metabolites in human health. The microbiota is defined as the aggregate of all bacteria, viruses, fungi, and archaea residing at various body interfaces, including the skin, oral cavity, intestinal lumen, and lungs. This additional “organ” of the human body contains approximately 150 times more genetic material than the human genome [4]. The composition of the gut microbiota is principally constituted by six phyla: The predominant bacterial phyla identified in the sample under investigation were Bacillota (Gram-positive), Bacteroidota (Gram-negative), Actinobacteria (Gram-positive), Proteobacteria (Gram-negative), Fusobacteria (Gram-negative), and Verrucomicrobia (Gram-negative). Notably, Bacillota and Bacteroidota were the most dominant [5].

Lipopolysaccharides (LPS) are major structural components of the cell wall in Gram-negative bacteria. They are glycolipids, composed of lipid A and a carbohydrate moiety. The toxic effects of LPS are attributed to the lipid A component, which binds to the Toll-like receptor 4 (TLR4), while their immunogenicity depends on the polysaccharide portion [6]. Not all lipopolysaccharides exert the same effects on the host. Some, such as those produced by Escherichia coli, are highly pathogenic, whereas others, like those secreted by Bacteroides species, exhibit immunomodulatory properties [7].

The intestinal epithelium comprises several protective layers, each employing distinct mechanisms to ensure optimal defense of the underlying tissues [8]. The outermost layer is constituted by intestinal alkaline phosphatase (IAP), which is secreted by enterocytes into the lumen. It has been demonstrated that IAP is capable of neutralising lipopolysaccharides (LPS) by dephosphorylating one of the two phosphate groups on lipid A. This process has been shown to reduce the toxicity of LPS by approximately 100-fold [9]. The second layer is the outer mucin layer, which is composed primarily of glycoproteins and water. This layer contains commensal bacteria that inhibit the penetration of pathogens into the inner mucin layer, which is tightly bound to the epithelial cells. The homeostasis of the mucin layers is dependent on the secretion of mucins by goblet cells and the utilisation of mucins by resident bacteria.

The intestinal epithelium is the subject of the subsequent zone, which is responsible for the regulation of substance entry into the body via transcellular and paracellular transport mechanisms. The utilisation of transcellular transport is conventionally employed for the conveyance of nutrients and electrolytes. Conversely, paracellular transport facilitates the movement of ions and solutes. The final layer of defence involves the local secretion of antimicrobial peptides with bactericidal properties by Paneth cells [10]. These peptides include α- and β-defensins. Under normal conditions, the bacterial population in the intestinal lumen may contain approximately 10–50 g of endotoxin; however, effective barrier functions prevent these molecules from entering systemic circulation [11]. Disruptions in the proteins regulating paracellular transport are commonly associated with the translocation of bacterial products and/or bacteria into the host. An alternative route of entry is lipid-associated transcellular transport, which becomes particularly relevant in the context of a high-fat diet [12].

Under physiological conditions, LPS are either undetectable in the circulation or present at concentrations of approximately 5–10 ng/L, which do not elicit a systemic response [11,13]. In the event of LPS entering the bloodstream, they are rapidly eliminated by Kupffer cells in the liver. A circulating concentration exceeding 20 ng/L is considered indicative of low-grade endotoxemia [14].

The primary mechanism by which LPS exert their biological effects involves binding to TLR4, leading to the induction of pro-inflammatory cytokines such as TNF-α, IL-1β, and IL-6, as well as the activation of NF-κB and subsequent production of IFN-β. Furthermore, it has been demonstrated that LPS, through the action of TLR4 signaling, can suppress the expression of proteins involved in the formation of tight junctions between epithelial cells. This, in turn, has been shown to compromise a key component of the host’s barrier defense system [15].

Beyond their immunological effects, LPS also directly influence thyroid gland function. It has been demonstrated that the substances in question have the capacity to stimulate thyroglobulin synthesis and enhance the functional activity of thyrocytes. In addition, they have been shown to modulate circulating thyroid hormone levels by selectively inhibiting type 1 deiodinase activity in peripheral tissues and activating type 2 deiodinase in the hypothalamus and pituitary gland [16].

The primary objective of this study was to assess circulating LPS concentrations and dietary patterns in patients with Hashimoto’s thyroiditis compared to healthy controls.

## 2. Materials and Methods

### 2.1. Study Design

The investigation was designed as a hospital-based case–control study and was conducted in accordance with the Declaration of Helsinki. Ethical approval was obtained from the Ethics Committee of the Medical University of Plovdiv (protocol code No. 4/04.05.2023 and date of approval 4 May 2023). Prior to enrolment, written informed consent was obtained from all participants.

Patients were recruited from the population of newly diagnosed cases of Hashimoto’s thyroiditis evaluated and treated at the Clinic of Endocrinology and Metabolic Diseases, UMHAT “St. George” Ltd., Plovdiv, Bulgaria. The control group comprised healthy volunteers who were matched for age and sex. Participants were eligible for inclusion if they met the following conditions: (1) clinically confirmed diagnosis of Hashimoto’s thyroiditis, established through characteristic laboratory findings (elevated TPOAb and/or TgAb, and thyroid function tests) and supportive thyroid ultrasound features; (2) ability to communicate in Bulgarian; (3) provision of written informed consent. Exclusion criteria: (1) current use of thyroid hormone replacement therapy or antithyroid medications, which could alter thyroid function or immune parameters; (2) history of thyroid surgery, including partial or total thyroidectomy; (3) use of medications influencing carbohydrate or lipid metabolism within the preceding six months; (4) presence of other endocrine disorders; (5) confirmed immunological or infectious diseases; (6) active oncological conditions.

### 2.2. Participant Recruitment and Grouping

Between May 2023 and November 2024, 130 individuals with suspected Hashimoto’s thyroiditis were screened for eligibility. Of these, 125 participants fulfilled the inclusion criteria and were enrolled in the study. Recruitment of HT patients and healthy controls was performed in parallel to ensure comparability between groups. The diagnosis of HT was confirmed through a comprehensive evaluation including clinical history, physical examination, thyroid ultrasound, and laboratory investigations of thyroid hormone profiles and autoantibody levels. Healthy controls were recruited from the same hospital catchment area and were matched to patients by age and sex.

A total of 105 newly diagnosed patients with Hashimoto’s thyroiditis (HT) were enrolled in the study, while 20 eligible individuals (16%) declined participation. Control participants were healthy individuals who met the same inclusion criteria and were recruited from the same hospital during routine outpatient consultations. In total, 86 eligible controls were identified; of these, 46 (53.5%) declined participation, 11 were excluded due to insufficient blood samples, and 4 were excluded because of incomplete questionnaire data. As five individuals from the initial screening phase were newly diagnosed with Hashimoto’s thyroiditis and transferred to the patient group, we additionally recruited five new control participants to maintain the intended sample size. Ultimately, 25 control subjects were included in the final analysis.

In accordance with thyroid hormone profiles, patients diagnosed with Hashimoto’s thyroiditis (n = 105) were stratified into the following subgroups: euthyroid (n = 61), tendency toward hypofunction (n = 34), subclinical hypothyroidism (n = 3), and overt hypothyroidism (n = 7). According to serum lipopolysaccharide (LPS) concentrations, participants were further categorized into ≤4 ng/kg and >4 ng/kg groups based on thresholds used in previous metabolic studies; however, this cut-off has not been validated for autoimmune thyroid diseases and is applied here in an exploratory manner [17,18]. Participants with low-titer TRAb positivity but without clinical or biochemical evidence of overt Graves’ disease or thyrotoxicosis were not excluded, as such findings may reflect TRAb-blocking activity and are compatible with hypothyroid presentations of autoimmune thyroid disease.

An assessment of dietary habits and nutrition beliefs was conducted utilising the KomPAN questionnaire (Dietary Habits and Nutrition Beliefs Questionnaire), a tool that has been validated and developed by the Polish Academy of Sciences [19,20]. The instrument utilised in this study evaluates the frequency of consumption of selected food groups, lifestyle factors, and nutrition-related attitudes. Responses were recorded on a standardised Likert-type scale, enabling quantitative analysis of dietary patterns. The questionnaire has been extensively utilised in Central and Eastern European populations, exhibiting commendable reliability and validity for nutritional epidemiology studies [21,22]. Dietary intake was assessed using the validated KomPAN questionnaire, which includes components on dietary habits, food frequency, consumption, nutrition beliefs, and lifestyle. The DH scale comprises 10 items, while the FFC scale includes 33 food groups assessed on a 6-point frequency scale. Following KomPAN methodology, frequency categories were converted into numerical values reflecting weekly consumption, and for analysis, intake was further classified as less than once per week or at least once per week. The Pro–Healthy Diet Index (pHDI) was calculated according to the KomPAN scoring protocol [23]. Only variables considered clinically relevant and likely to discriminate between participants were included in the pattern analysis.

Following the process of informed consent, both patients and the control group completed the questionnaire during a single visit to the assessment centre. All participants were subjected to a comprehensive medical history interview, a physical examination, and a thyroid ultrasound performed by a certified endocrinologist [24].

### 2.3. Ethical Considerations

Prior to the statistical analysis, all participant data were anonymised and coded in accordance with the European Union’s General Data Protection Regulation (GDPR). The study was conducted in accordance with the ethical principles set out in the Declaration of Helsinki for medical research involving human subjects.

Ethical approval was obtained from the Ethics Committee of the Medical University of Plovdiv (Protocol No. 4/04.05.2023). The research was conducted in accordance with Bulgarian national legislation and regulations governing clinical and scientific studies involving human participants, as well as the internationally recognised standards of Good Clinical Practice (GCP). Prior to enrolment, written informed consent was obtained from all participants.

### 2.4. Sample Collection

The procurement of biological samples occurred concurrently with routine clinical blood draws between 7:30 and 9:00 a.m., following a standardized 12-h overnight fasting period. No additional invasive procedures were required for sample acquisition.

Blood specimens were collected in tubes containing K_3_-EDTA (tripotassium ethylene-diaminetetraacetic acid) anticoagulant. Immediately following collection, the tubes were placed in a centrifuge, and the separated plasma was stored at −70 °C until analysis. Samples collected without the use of an anticoagulant were permitted to clot at ambient temperature for a period of 30 min. Following this, the samples were subjected to a process of centrifugation. Thereafter, the serum that resulted was divided into smaller containers (aliquots) and frozen at a temperature of −70 °C until further examination.

### 2.5. Laboratory Analysis

The measurement of thyroid hormones (TSH, fT3, fT4) and autoantibodies (antiTG, antiTPO, TRAB) was conducted using a chemiluminescent immunoassay on a Mindray analyzer (Mindray Bio-Medical Electronics Co., Ltd., Shenzhen, China). The determination of serum concentrations of lipopolysaccharides (LPS) was conducted by utilising commercial ELISA kits (Abbexa Ltd., Cambridge, UK). The sensitivity of the assay was found to be less than 3.5 pg/mL. The intra- and inter-assay precision, as declared, was found to be less than 9%. Absorbance readings were performed using a TECAN Sunrise ELISA reader (TECAN, Männedorf, Switzerland) at 450 nm.

### 2.6. Statistical Analysis

The data analysis was conducted utilising IBM SPSS Statistics, version 28.0 for Windows (SPSS Inc., Chicago, IL, USA). The continuous variables were expressed in terms of mean (M) and standard deviation (SD) to facilitate the analysis of the descriptive statistics. The healthy controls were intended to be matched to HT patients by sex and age; however, residual differences in age and BMI remained and were considered in the interpretation of the findings. Despite the matching process, a slight yet statistically significant disparity in age remained between the groups. This residual imbalance was accounted for in the statistical analyses. The Mann–Whitney U test was employed to compare the differences between groups for continuous variables, whilst the chi-square test was utilised for categorical data. Pearson correlation analysis was conducted in order to examine the associations between serum LPS concentrations, thyroid hormone profiles, and antibody levels. A case–control comparison was conducted with the objective of identifying food groups that exhibited significantly disparate consumption frequencies between patients diagnosed with hypothyroidism (HT) and healthy controls. Statistical significance was defined as *p* < 0.05. Post-hoc power analysis, based on the observed effect size for circulating LPS (Cohen’s d = 0.95) and the group sizes (105 vs. 25), demonstrated statistical power of 0.93 at α = 0.05.

## 3. Results

### Participant Characteristics

The final analysis comprised 105 patients with newly diagnosed Hashimoto’s thyroiditis and 25 healthy controls. Within the HT cohort, 82 participants were female (63.1%) and 23 were male (17.9%). In the control group, 13 subjects were female (52.0%) and 12 were male (48.0%). A statistically significant age difference was observed (*p* = 0.034), although the mean ages of the groups remained relatively close (see Table 1 for details).

The mean values of thyroid hormone parameters, thyroid-specific autoantibodies, and LPS are summarised in Table 2.

The following reference ranges were utilised for thyroid hormones and autoantibody levels: The following parameters were measured: TSH (0.34–5.1 mU/L), fT3 (3.8–6.3 pmol/L), fT4 (11.0–23.0 pmol/L), anti-TPO (0–9 IU/mL), and anti-Tg (0–4 IU/mL).

**Figure 1:**
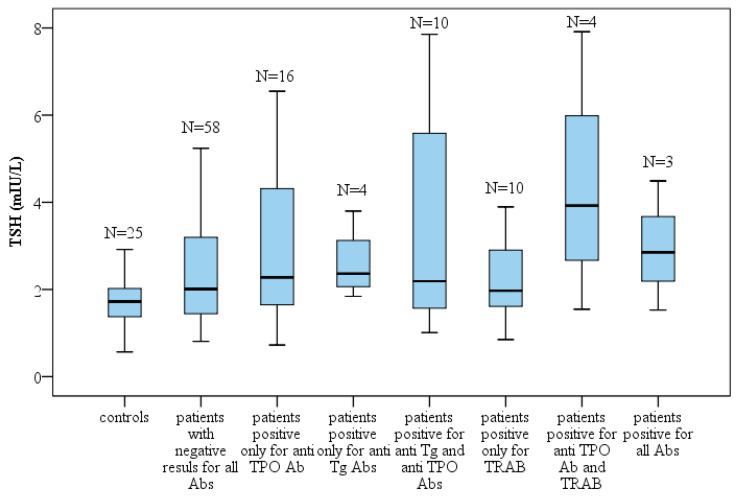
Distribution of TSH levels across groups based on autoantibody status. Box-plot diagram, Kruskal–Wallis test; N = number of participants.

Patients with elevated anti-TPO and TRAb (Thyrotropin Receptor Antibodies) levels exhibited the highest TSH concentrations, followed by those with elevations in all three types of thyroid autoantibodies (see Figure 1). Within the seronegative subgroup, 35 individuals were classified as euthyroid, 21 exhibited a tendency toward developing hypothyroidism, and single cases of subclinical and overt hypothyroidism were also observed.

The distribution of mean values for thyroid hormones, fT4 and fT3, revealed a discernible trend, primarily evident in fT4 levels.

**Figure 2:**
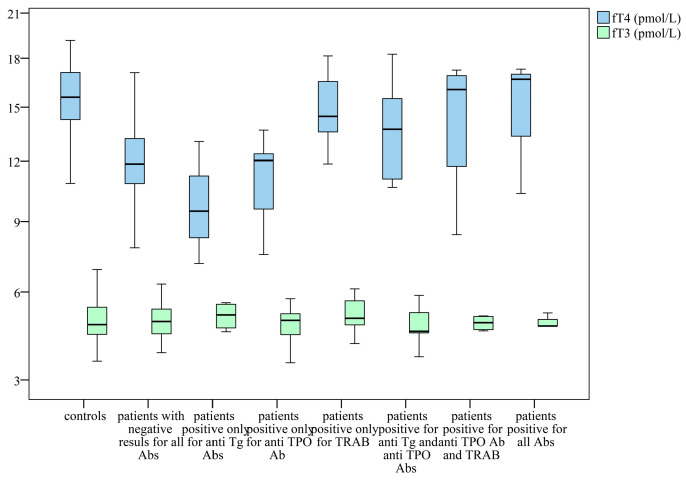
Distribution of fT3 and fT4 levels across groups based on autoantibody status. Box-plot diagram, Kruskal–Wallis test.

Patients with elevated anti-Tg autoantibody levels exhibited the lowest mean concentrations of fT4. However, the fT3 levels of the study group remained comparable to those observed in the control group (see Figure 2).

**Figure 3:**
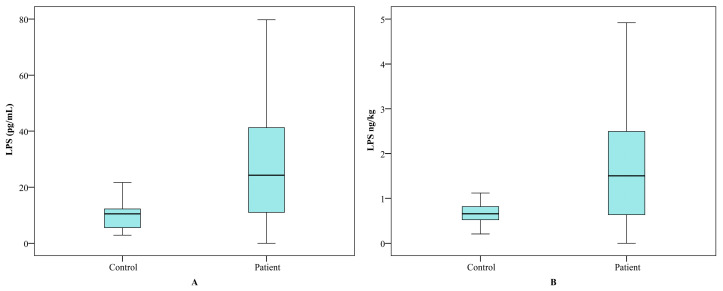
Distribution of actual (pg/mL) (**A**) and normalized (ng/kg) (**B**) LPS values in patients (n = 105) and controls (n = 25). Box-plot diagram, Kruskal–Wallis test.

Normalised LPS values were calculated using Nadler’s formula [21], which considers body weight and circulating blood volume. The original and adjusted distributions of LPS concentrations are presented in Figure 3.

**Figure 4:**
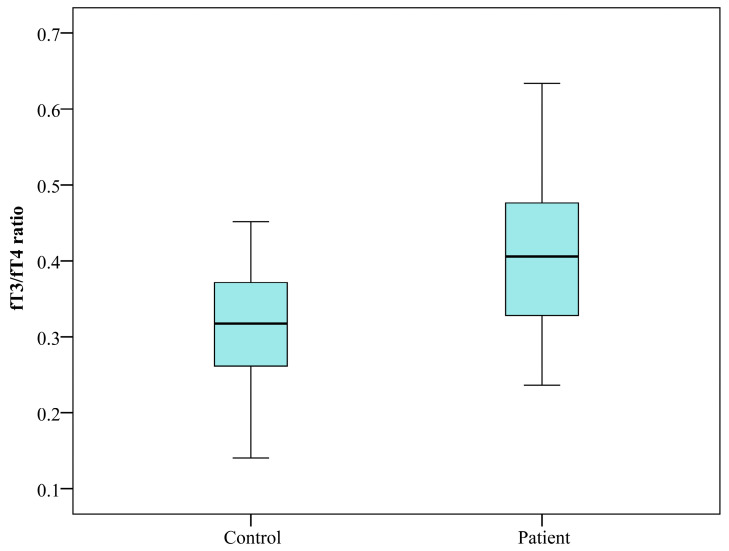
fT3/fT4 ratio in patient (n = 105) and control (n = 25) groups. Box-plot diagram, Kruskal–Wallis test.

Deiodinases are selenoprotein enzymes that regulate thyroid hormone activity by catalyzing the activation or inactivation of thyroxine (T4) and triiodothyronine (T3) in human tissues [25]. One approach to assessing deiodinase activity in the human body is by calculating the fT3/fT4 ratio [26]. As demonstrated in Figure 4, the patient group exhibited a considerably elevated ratio in comparison to the control group (*p* = 0.001).

**Figure 5:**
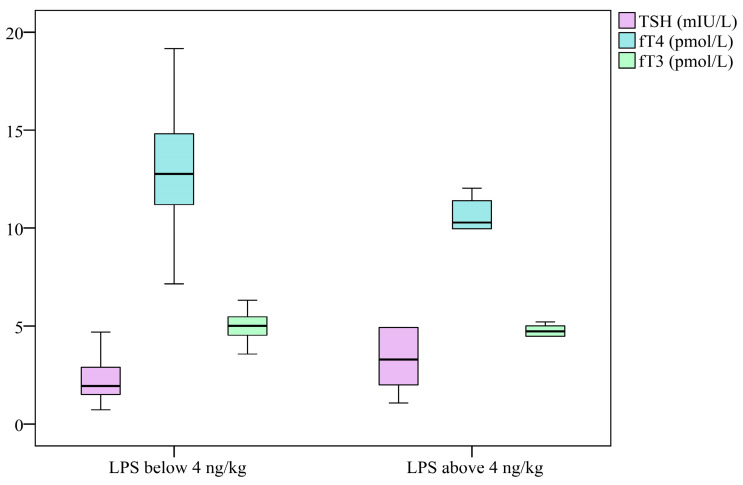
Distribution of TSH, fT3 and fT4 results in groups according to LPS levels—below (n = 99) and above (n = 6) 4 ng/kg. Box-plot diagram, Kruskal–Wallis test.

Despite the mean LPS concentrations in the control group being significantly lower than those in the patient group (*p* = 0.02), values in both groups remained within the range of up to 4 ng/kg body weight. However, the distribution revealed that some patients exhibited LPS levels exceeding this threshold. The stratification based on the 4 ng/kg threshold should be interpreted cautiously, as this cut-off has not been validated for autoimmune thyroid diseases. The findings of the present study indicated a statistically significant correlation between the concentrations of LPS and the fT3/fT4 ratio (*r* = 0.247, *p* = 0.006). In patients with LPS levels exceeding 4 ng/kg body weight, lower mean values of both fT3 and fT4 were observed, with fT4 showing a statistically significant reduction (*p* = 0.029). A negative correlation was also identified between fT4 and LPS levels (*r* = −0.314, *p* < 0.001) (see Figure 5). It appears that increased intestinal permeability is associated with the activation of the autoimmune process in the thyroid gland. In patients diagnosed with Graves’ disease, levels of LPS were found to be elevated in comparison to the control group. Furthermore, increased levels of LPS have been associated with more severe cases of hyperthyroidism, which is characterised by elevated fT3 and fT4 levels, augmented TRAb levels, and diminished TSH [27].

**Figure 6:**
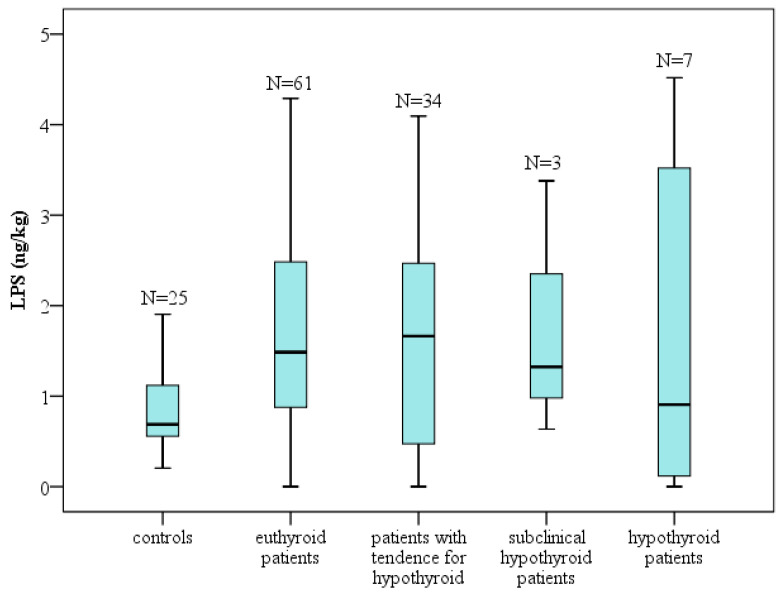
Distribution of LPS values in groups according to Thyroid status. Box-plot diagram, Kruskal–Wallis test; N = number of participants.

In the case of Hashimoto’s thyroiditis, the autoimmune process is initiated, resulting in the exhaustion of glandular capacity and the subsequent development of hypothyroidism. In the present study, the highest mean levels of LPS were observed in patients with pronounced thyroid hypofunction (see Figure 6).

As illustrated in Table 3, a comparison has been made between the frequency of weekly consumption (times/week) of selected food products among patients diagnosed with Hashimoto’s thyroiditis (HT) and that of a control group of individuals deemed to be healthy [28]. A comparative analysis was conducted to ascertain disparities among the groups with regard to their dietary intake patterns, with the objective of identifying those food categories that exhibited significantly divergent intake patterns.

Patients diagnosed with Hashimoto’s thyroiditis (HT) reported a higher frequency of consumption of rice, vegetable oils, fruits, and vegetables when compared to healthy controls. In contrast, the control group demonstrated a higher intake of wholemeal and other wholegrain products (including oats), fermented milk beverages, fresh cheese products, meat and fish, eggs, beans, and fruit juices.

Significant positive correlations were observed for wholemeal products (ρ = 0.178, *p* = 0.043), oats/wholegrain products (ρ = 0.352, *p* = 0.001), fermented milk beverages (ρ = 0.195, *p* = 0.026), fresh cheese products (ρ = 0.265, *p* = 0.002), eggs (ρ = 0.177, *p* = 0.031), legumes (ρ = 0.177, *p* = 0.031), fruit juices (ρ = 0.286, *p* = 0.001), and the pro-Healthy Diet Index (pHDI) (ρ = 0.276, *p* = 0.001), indicating that higher intake of these foods was associated with healthier dietary patterns.

## 4. Discussion

Hashimoto’s thyroiditis is a chronic autoimmune disorder that often eludes early diagnosis due to its asymptomatic or nonspecific clinical presentation, particularly in individuals with preserved thyroid function. It has been established that a significant proportion of patients who have been newly diagnosed may not exhibit elevated circulating autoantibodies. This has the effect of further complicating detection. Common symptoms such as fatigue, mood disturbances, and weight gain are typically subtle and nonspecific, frequently resulting in incidental diagnosis during routine medical evaluations [29]. In accordance with this observation, a subsequent re-evaluation of the clinical and instrumental data by a certified endocrinologist resulted in the reclassification of five participants initially categorised as controls in our study as HT patients.

The predominance of euthyroid status among participants was anticipated, given that the inclusion criteria targeted newly diagnosed or untreated individuals. It is noteworthy that almost half of the HT patients did not demonstrate elevated autoantibodies at the time of testing, thereby underscoring the limitations of serological markers in isolation as a diagnostic tool.

Lipopolysaccharides (LPS) are structural components of Gram-negative bacterial cell walls. They have been shown to be potent activators of the Toll-like receptor 4 (TLR4) signalling cascade, inducing the release of pro-inflammatory cytokines such as TNF-α, IL-6, IL-1, and IL-1β [30]. Furthermore, LPS have been implicated in cardiovascular pathology via direct activation of the NLRP3 inflammasome [31,32].

The present findings may indicate a potential mechanistic association between elevated serum LPS levels and HT, although this relationship cannot be considered causal based on the current data. Patients with elevated anti-TPO and TRAb antibody titres exhibited the highest TSH concentrations, suggesting a more advanced stage of autoimmune involvement. It is also noteworthy that individuals with elevated anti-TG antibodies demonstrated the lowest mean fT4 levels, while fT3 remained comparable to the control group—a pattern that might suggest compensatory peripheral deiodinase activity.

Within the context of this study, introducing of a cut-off value of 4 ng/kg body weight for stratifying LPS levels appears to delineate groups with differing thyroid hormone profiles. Although this threshold is exploratory, individuals with higher LPS concentrations tended to show lower fT3 and fT4 levels, with the reduction in fT4 reaching statistical significance (*p* = 0.029). These patterns may reflect underlying immunometabolic or endocrine differences associated with elevated LPS exposure; however, they should be interpreted cautiously, as the observed associations do not establish causality and may instead capture broader variability in immune activation or metabolic status. The fT3/fT4 ratio was found to be significantly elevated in patients diagnosed with HT (*p* = 0.001), indicative of altered deiodinase regulation. Further correlation analysis demonstrated a positive association between LPS and the fT3/fT4 ratio (*r* = 0.247, *p* = 0.006), and a negative association between LPS and fT4 (*r* = −0.314, *p* < 0.001).

The present findings are consistent with existing evidence and suggest the possibility that increased intestinal permeability and systemic endotoxemia may be involved in the initiation or amplification of autoimmune thyroid dysfunction. Elevated circulating LPS levels have also been observed in Graves’ disease, where they have been reported to be associated with more pronounced hyperthyroid states. While these patterns may offer some indication, their interpretation must be approached with caution, as the available data do not establish a direct causal relationship [33].

BMI is widely recognised as an indicator of overall adiposity and metabolic status, and higher BMI is frequently associated with low-grade systemic inflammation, altered adipokine secretion, and metabolic stress, all of which may influence autoimmune processes [34]. Previous studies have reported that individuals with Hashimoto’s thyroiditis (HT) often present with higher BMI compared with euthyroid controls, suggesting a bidirectional relationship between thyroid dysfunction, metabolic regulation, and body composition [35]. This pattern was also observed in our study, where BMI tended to be higher among HT patients than among healthy controls, further supporting the relevance of metabolic status in the context of HT.

In parallel with these metabolic considerations, recent research has highlighted the importance of dietary patterns and specific dietary components in the aetiology and clinical expression of HT [36,37,38]. The present study was conducted with the objective of investigating the influence of diet on the development of HT disease. To this end, a series of analyses were conducted in order to identify food groups that differed between HT patients and the control group. The present study revealed that patients suffering from Hashimoto’s disease exhibited a reduced tendency to consume whole grains, dairy products, fresh cheese products, meat, fish and legumes when compared to a control group of healthy individuals. Despite the absence of a definitive dietary intervention for the management of Hashimoto’s disease, the adoption of a nutrient dense, anti-inflammatory diet has been demonstrated to be a promising approach for effective symptom control. As demonstrated in the research by Kaličanin et al., higher intake of animal fats, processed meats, and nuts was observed among HT patients. In contrast, the control group reported greater consumption of red meat, soft drinks, whole grains, vegetable oils, olive oil, alcoholic beverages, fatty fish, and fruits [39]. In addition, other studies have indicated that individuals diagnosed with HT tend to consume greater quantities of animal-based foods, while those considered to be healthy favour plant-based diets comprising legumes, fruits, vegetables, and nuts [40].

The most significant result of our study was the observation of a substantial decrease in the consumption of whole grains, fermented milk beverages and fresh cheese products among HT patients in comparison to the control group.

In our study, the Pro-Healthy Diet Index (pHDI) was significantly lower among individuals with Hashimoto’s thyroiditis (HT) compared with healthy controls (3.94 vs. 5.34, *p* = 0.001), suggesting the presence of less favourable dietary patterns in the HT group. Although such differences may hypothetically contribute to impaired gut barrier integrity and increased translocation of lipopolysaccharides (LPS), these interpretations should be made with caution. The findings should be viewed as exploratory and hypothesis-generating.

The results of this study provide support for a multifactorial model, in which dietary imbalance is shown to promote intestinal permeability, elevate circulating LPS concentrations, and trigger autoimmune activity within the thyroid gland. These observations underscore the pivotal role of diet as a modifiable risk factor in the pathogenesis of HT. It is recommended that future research endeavours focus on further investigating the therapeutic potential of dietary modification and microbiota-targeted interventions in mitigating disease onset and progression.

The present study is subject to several limitations. Firstly, the case–control design does not permit the drawing of causal inferences regarding the relationships between circulating LPS, thyroid function, and dietary patterns. Secondly, it is important to note that all biochemical measurements—including those of LPS, thyroid hormones, and autoantibodies—were obtained at a single time point. This may not fully reflect temporal variability or the dynamic course of autoimmune thyroiditis. Thirdly, although the overall sample size provided adequate statistical power to detect medium-to-large differences in LPS concentrations, the control group was relatively small, potentially limiting the detection of subtler associations. In the fourth instance, the dietary intake of the subjects was the subject of assessment. This was achieved by means of a food frequency questionnaire which was self-reported. It should be noted, however, that this method is prone to a number of potential problems. These include recall bias, misclassification, and the lack of portion size estimation. Furthermore, the measurement of LPS relied on an ELISA-based assay. Despite its widespread use in research, this method may be affected by limited analytical specificity and potential interference from circulating lipoproteins or endotoxin-binding proteins. It is imperative that these methodological considerations be taken into account when interpreting the results. Finally, unmeasured factors such as gut microbiota composition, intestinal permeability markers, medication use, and lifestyle variables may have influenced both LPS levels and thyroid function, thereby constraining the mechanistic interpretation of the findings.

## 5. Conclusions

This study shows that HT patients exhibit higher LPS levels compared with controls; however, these associations are exploratory and should be interpreted cautiously due to residual confounding and the observational design. The observed hormonal and immunological alterations—including increased thyroid-stimulating hormone (TSH) concentrations and disrupted deiodinase activity—may be compatible with a potential contributory role of LPS in disease progression.

Moreover, significant variations in dietary habits between HT patients and healthy controls highlight the potential influence of nutrition on intestinal permeability and immune activation. The findings, taken collectively, underscore the significance of incorporating dietary strategies and microbiota-targeted interventions into the prevention and management of autoimmune thyroid disorders. It is recommended that future longitudinal and interventional studies are conducted to clarify causal relationships and evaluate the therapeutic efficacy of such approaches.

## Figures and Tables

**Table 1:** Descriptive characteristics of the study participants (N = 130).

Characteristics	Patients with HT (n = 105)	Controls (n = 25)	*p*
**Gender, n (%)**			
Female	82 (78.1%)	13 (52.0%)	0.008 *
Male	23 (21.9%)	12 (48.0%)	
**Residence, n (%)**			
Urban	94 (89.5%)	23 (92.0%)	0.711
Rural	11 (10.5%)	2 (8.0%)	
**Educational level, n (%)**			
primary	5 (4.8%)	0 (0%)	0.549
secondary	33 (31.4%)	9 (36.0%)	
colleges	3 (2.9%)	2 (8.0%)	
bachelor’s degree	23 (21.9%)	4 (16.0%)	
master’s degree	41 (39.0%)	10 (40.0%)	
**Smoking**			
no	77 (80.9%)	20 (80.0%)	0.491
yes	28 (29.4%)	5 (20.0%)	
**Alcohol use**			
no	37 (35.2%)	8 (32.0%)	0.145
yes	68 (64.8%)	17 (68.0%)	
**Physical activity level**			
Low	48 (45.7%)	9 (36.0%)	0.634
Moderate	50 (47.6%)	16 (64.0%)	
High	7 (6.7%)	0 (0%)	
	**mean (SD)**	**mean (SD)**	
**Age (years)**	39.29 (1.66)	32.87 (3.10)	0.034 *
**BMI (kg/m^2^)**	28.10 (0.74)	24.69 (1.13)	0.064

BMI (body mass index), *p* value < 0.05, Mann–Whitney-U test, χ^2^-test, * *p*—*p* value < 0.05

**Table 2:** Mean values of thyroid hormones, autoantibodies and LPS.

Variable	HT Cases			Controls			*p*
	Mean	Min–Max	SD	Mean	Min–Max	SD	
TSH, mU/L	2.5	0.7–6.5	1.29	1.7	0.6–2.9	0.65	0.017 *
fT4, pmol/L	12.5	6.9–18.3	2.51	15.7	10.9–22.4	2.94	<0.001 *
fT3, pmol/L	4.9	3.5–6.3	0.60	4.9	3.1–6.9	0.89	0.832
antiTg, IU/mL	2.5	0.1–22	3.60	0.8	0.8–1.0	0.04	0.018 *
antiTPO, IU/mL	6.0	0.1–93	17.30	0.2	0.2–0.5	0.09	<0.001 *
LPS, pg/mL	27.3	0.1–79.8	19.40	10.3	2.9–21.6	4.98	<0.001 *

TSH, thyroid-stimulating hormone; fT3, free triiodothyronine; fT4, free thyroxine; antiTg, thyroglobulin autoantibodies; aniTPO, thyroid peroxidase autoantibodies; LPS, Lipopolysaccharides; * *p*—*p* value < 0.05, Mann–Whitney-U test.

**Table 3:** Frequency of consumption of selected food groups at “least once per week” and Pro-Healthy Diet Index (pHDI) among Cases and Controls.

Parameter	HT Cases n (%)	Controls n (%)	*p* (Group Comp.)	Spearman’s ρ	*p* (Correl.)
White bread/bakery	75 (71.4%)	19 (76%)	0.426	−0.028	0.750
Wholemeal	61 (58.1%)	20 (80%)	0.033 *	+0.178	0.043 *
Rice	78 (74.3%)	15 (60%)	0.121	−0.040	0.652
Oats/wholegrain products	27 (25.7%)	17 (68%)	0.000 *	+0.352	0.001 *
Butter	46 (43.8%)	12 (48%)	0.437	−0.018	0.827
Vegetable oils	23 (21.9%)	4 (16%)	0.364	−0.016	0.852
Fermented milk beverages	80 (76.2%)	24 (96%)	0.018 *	+0.195	0.026 *
Fresh cheese products	86 (81.9%)	24 (96%)	0.012 *	+0.265	0.002 *
Cold meats	68 (64.8%)	17 (68%)	0.447	−0.076	0.337
Red Meats	73 (69.5%)	19 (76%)	0.354	−0.013	0.874
White Meats	81 (77.1%)	21 (84%)	0.326	+0.020	0.800
Fish	40(38.1%)	13 (52%)	0.148	+0.119	0.145
Eggs	78 (74.3%)	21 (84%)	0.227	+0.177	0.031 *
Legumes	63 (60%)	19 (76%)	0.102	+0.177	0.031 *
Beans	63 (60%)	19 (76%)	0.102	+0.102	+0.158
Fruit	95 (90.5%)	22 (88%)	0.474	−0.076	0.337
Vegetables	102 (97.1%)	24 (96%)	0.579	+0.579	+0.124
Fruit juices	20 (19%)	12 (48%)	0.078	+0.286	0.001 *
pro-Healthy-Diet-Index (pHDI) Mean (SD) (range 0–20)	3.94 (1.95)	5.34 (2.04)	0.001 *	+0.276	0.001 *

*p*–*p* value < 0.05, χ^2^-test, Spearman correlation, * *p*—*p* value < 0.05.

## Data Availability

The data presented in this study are available on request from the corresponding author due to the sensitive nature of the clinical information.

## Abbreviations

The following abbreviations are used in this manuscript:

HT	Hashimoto’s thyroiditis
GD	Graves’ disease
LPS	Lipopolysaccharides
IAP	intestinal alkaline phosphatase
KomPAN	Dietary Habits and Nutrition Beliefs Questionnaire
GDPR	General Data Protection Regulation
GCP	Good Clinical Practice
K_3_-EDTA	Tripotassium Ethylenediaminetetraacetic Acid
ELISA	Enzyme-Linked Immunosorbent Assay
TSH	Thyroid-Stimulating Hormone
fT3	Free Triiodothyronine
fT4	Free Thyroxine
antiTg	Thyroglobulin Autoantibodies
aniTPO	Thyroid Peroxidase Autoantibodies
TRAb	Thyrotropin Receptor Antibodies
